# Primary care of children and young people with asthma during the COVID-19 era

**DOI:** 10.3399/bjgp20X713165

**Published:** 2020-10-30

**Authors:** Hanna Creese, David Taylor-Robinson, Sejal Saglani, Sonia Saxena

**Affiliations:** School of Public Health, Imperial College London, London.; Department of Public Health and Policy, University of Liverpool, Liverpool.; National Heart and Lung Institute, Imperial College London, London.; School of Public Health, Imperial College London, London.

## INTRODUCTION

Around 1.1 million children and young people (CYP) currently receive treatment for asthma in the UK.^1^ The UK performs poorly compared with other European countries in children’s outcomes of asthma management and has had among the highest number of reported asthma deaths in Europe since 1998.^2^ Here we evaluate evidence of the impact of COVID-19 on CYP with asthma and consider what actions GPs can take to protect these children from serious harm.

## ARE CHILDREN WITH ASTHMA AT INCREASED RISK OF COVID-19 INFECTION?

There is no strong evidence that CYP with asthma are more susceptible to COVID-19 infection than the general population, although children with long-term conditions are under-represented in epidemiological studies.^3^ In fact, there is even some evidence that, as CYP with atopic asthma tend to have lower angiotensin-converting enzyme 2 (ACE2) expression, they are potentially less susceptible to COVID-19 infection.^4^

Across Europe, the overall incidence of COVID-19 cases for CYP is 1.8%: 1.1% for children aged <10 years and 2.5% for young people aged 10–19 years.^5^ However, incidence is variable and highly dependent on exposure. Fortunately, most CYP with COVID-19 usually experience an asymptomatic infection or mild self-limiting illness, with only very few experiencing the hyperinflammatory response, Paediatric Inflammatory Multisystem Syndrome (PIMS).^6^ Hence, CYP with asthma are more likely to experience poor health because of their underlying long-term condition rather than COVID-19 infection itself.

Viral infections worsen asthma symptoms. Viruses are detected in up to 80% of asthma exacerbations and annual seasonal strains of coronavirus are associated with 8.4% of asthma exacerbations in CYP.^6^ An asthma exacerbation would require a child to attend, and potentially be admitted to, a healthcare setting that may carry a small additional risk of exposure to COVID-19.

Children are less than half as likely to transmit COVID-19 compared with adults,^7^ but transmission will inevitably increase with the reopening of schools. Previous years have shown winter peaks in flu and respiratory infections, and hospitalisations among children with asthma after schools open in the autumn. However, if this expected spike is concurrent with a second wave of the COVID-19 pandemic, paediatric wards could be at reduced capacity, with staff and space deployed for adult COVID-19 patients instead. Children’s vaccinations fell earlier this year^8^ and health professionals fear the flu vaccine uptake may be similarly poor, leading to greater than expected flu cases.

## ARE CHILDREN WITH ASTHMA AT RISK OF MORE SEVERE COVID-19 INFECTION?

A European study has shown that, if CYP with COVID-19 have a pre-existing medical condition or a lower respiratory tract infection, they are up to 10 times more likely to require an intensive care admission.^3^ Of the 1.1 million CYP receiving treatment for asthma in the UK (9% of all CYP), around 44 000 CYP with severe asthma (4% of the total receiving treatment)^9^ were advised to shield for 12 weeks because of COVID-19 alongside those clinically vulnerable.

Symptoms of COVID-19 in CYP are less severe compared with adults but the evidence in CYP with asthma is mixed. Inhaled corticosteroids (ICS) prescribed for asthma could curb the inflammatory response in patients if they go on to develop COVID-19 infection. In vitro pre-treatment shows inhibitory effects of corticosteroids on coronavirus^10^ but there is currently no convincing evidence of this in humans. This would also contrast with earlier reports that corticosteroid use in patients with Middle East respiratory syndrome coronavirus led to prolonged viral replication and increased risk of mechanical ventilation.^11^

Initial international guidance advised that those with asthma were clinically extremely vulnerable.^12^ Yet, data from China and South Korea indicate that asthma is not a significant comorbidity for COVID-19 adults, and there is limited data on children to infer conclusions. Only 3% of CYP in China have asthma,^13^ compared with three times that in the UK.^1^ The low prevalence of asthma in China indicates that data are not generalisable to European children.

## HOW HAS CHILDREN’S ASTHMA BEEN AFFECTED BY COVID-19 CONTAINMENT MEASURES?

Overall, there were fewer severe asthma presentations in CYP to emergency departments,^14^ amid falls of up to 90% in CYP’s attendance to emergency departments during lockdown. The most likely explanation is that there have been fewer triggers from respiratory infection associated with school closures and social distancing. It is also likely that caregivers’ concern for children with asthma has driven better adherence to preventive medication.

To contain the global spread of COVID-19, border control measures and travel restrictions were implemented in numerous countries. Subsequent reductions in air and road traffic and industrial activity led to the reduction in some air pollutants across the UK, such as nitrogen dioxide and particulate matter. Consequently, a reduction in air pollution may have protected children from the worsening of asthma symptoms associated with these types of air pollution.

However, pre-existing health inequalities are stark in childhood asthma whereby children in the most deprived areas are twice as likely to suffer an emergency admission for asthma compared with those from the least deprived areas.^15^ Prolonged exposure to poor housing, indoor allergens, and passive smoke during months of protracted homestay may have led to further widening of asthma inequalities in CYP, as all these triggers are more common in households in deprived areas.

## HOW CAN GPs SUPPORT CHILDREN AND FAMILIES WITH ASTHMA DURING THE COVID-19 PANDEMIC?

Schools have reopened, social distancing rules are relaxed, and we are now heading into the flu season. Consequently, it is crucial that GPs are especially vigilant to health contacts from families of children with asthma. Remote assessment over the phone or video is challenging, with measurement of breathlessness unreliable, and parents and caregivers may report symptoms selectively. Therefore, poor asthma control may be missed, and early symptoms of COVID-19 in children may be misinterpreted as asthma symptoms and vice versa. All initial assessments, whether face-to-face or remote, should be accompanied by safety-netting advice on how to seek help in the event of deterioration, and education about the importance of preventive care.

GPs should have a low threshold for offering face-to-face assessment for CYP with acute asthma symptoms to assess severity and the need for onwards referral. Of concern are functional difficulties in feeding, speaking, and the work of breathing. Examination allows oxygen saturation and essential observations such as pulse, respiratory rate, and auscultation for wheeze.

There is debate around the use of nebulisers, with some arguing that their use should be discouraged unless essential as they aerosolise and increase the risk of contagion,^16^ and, instead, metered dose inhalers should be recommended in healthcare settings and in the home. However, the National Institute for Health and Care Excellence guidance on this states that patients can continue to use their nebuliser as the aerosol comes from the fluid in the nebuliser chamber and will not carry virus particles from the patient.^17^ There is no evidence of impaired immune response to COVID-19 in CYP with asthma being treated with biologic agents.

Asthma reviews to optimise preventive care and adherence are all the more important during this pandemic. Follow-up care should include health education to reduce asthma triggers including passive smoke exposure, assessment of inhaler technique, and device checks. Primary care teams should ensure Personal Asthma Action Plans (PAAP) are up to date and advise patients to adhere to guidelines to avoid any exacerbation. There should be active follow-up of patients requiring a PAAP who have not yet received one.

Diary alerts should be set up on GP systems to follow up all presentations of asthma exacerbation within 48 hours, particularly for those children who have attended emergency departments and to ensure regular reviews are scheduled, including follow-up of children who miss vaccinations. GPs need to ensure that CYP’s vaccines are up to date, especially for those with asthma. Integrated care pathways that allow rapid response and telephone advice from paediatricians can support GPs to keep CYP with asthma safe in the community and greatly reduce the risk of recurrence. Clinical commissioning groups should ensure these pathways are streamlined and communicated clearly to all primary care teams.

As health inequalities are largely driven by social determinants, solutions to asthma inequalities in CYP lie beyond the consulting room. Primary care teams play an important role in addressing inequalities by taking a whole-family approach to the care of CYP and encouraging greater connectivity between health professionals, social care, schools, and the community. While it is critical to improve quality of health care, COVID-19 has shown us that long-term investment in improving the quality and safety of the environments in which children live, play, and learn would benefit the health of all CYP.

## References

[1] Asthma UK (2020). Asthma facts and statistics.

[2] Shah R, Hagell A, Cheung R (2019). International comparisons of health and wellbeing in adolescence and early adulthood.

[3] Götzinger F, Santiago-García B, Noguera-Julián A (2020). COVID-19 in children and adolescents in Europe: a multinational, multicentre cohort study. Lancet Child Adolesc Health.

[4] Jackson DJ, Busse WW, Bacharier LB (2020). Association of respiratory allergy, asthma, and expression of the SARS-CoV-2 receptor ACE2. J Allergy Clin Immunol.

[5] De Luca CD, Esposito E, Cristiani L (2020). Covid-19 in children: a brief overview after three months experience. Paediatr Respir Rev.

[6] Zheng XY, Xu YJ, Guan WJ, Lin LF (2018). Regional, age and respiratory-secretion-specific prevalence of respiratory viruses associated with asthma exacerbation: a literature review. Arch Virol.

[7] Viner RM, Russell SJ, Croker H (2020). School closure and management practices during coronavirus outbreaks including COVID-19: a rapid systematic review. Lancet Child Adolesc Health.

[8] Saxena S, Skirrow H, Bedford H (2020). Routine vaccination during covid-19 pandemic response. BMJ.

[9] Pharmaceutical Services Negotiating Committee (PSNC) (2018). Essential facts, stats and quotes relating to asthma.

[10] Yamaya M, Nishimura H, Deng X (2020). Inhibitory effects of glycopyrronium, formoterol, and budesonide on coronavirus HCoV-229E replication and cytokine production by primary cultures of human nasal and tracheal epithelial cells. Respir Investig.

[11] Arabi YM, Mandourah Y, Al-Hameed F (2018). Corticosteroid therapy for critically ill patients with Middle East respiratory syndrome. Am J Respir Crit Care Med.

[12] Centers for Disease Control and Prevention (2020). People with asthma.

[13] Huang K, Yang T, Xu J (2019). Prevalence, risk factors, and management of asthma in China: a national cross-sectional study. Lancet.

[14] Roland D, Harwood R, Bishop N (2020). Children’s emergency presentations during the COVID-19 pandemic. Lancet Child Adolesc Health.

[15] Kossarova L, Cheung R, Hargreaves D, Keeble E (2017). Admissions of inequality: emergency hospital use for children and young people.

[16] van Doremalen N, Bushmaker T, Morris DH (2020). Aerosol and surface stability of SARS-CoV-2 as compared with SARS-CoV-1. N Engl J Med.

[17] National Institute for Health and Care Excellence (2020). COVID-19 rapid guideline: severe asthma NG166.

